# Design, Fabrication, and Testing of a Monolithically Integrated Tri-Axis High-Shock Accelerometer in Single (111)-Silicon Wafer

**DOI:** 10.3390/mi10040227

**Published:** 2019-03-29

**Authors:** Shengran Cai, Wei Li, Hongshuo Zou, Haifei Bao, Kun Zhang, Jiachou Wang, Zhaohui Song, Xinxin Li

**Affiliations:** 1 State Key Laboratory of Transducer Technology, Shanghai Institute of Microsystem and Information Technology, Chinese Academy of Sciences, Shanghai 200050, China; caishr@shanghaitech.edu.cn (S.C.); hszou@mail.sim.ac.cn (H.Z.); baohf@mail.sim.ac.cn (H.B.); zhangkun@mail.sim.ac.cn (K.Z.); jiatao-wang@mail.sim.ac.cn (J.W.); zhaohuisong@mail.sim.ac.cn (Z.S.); 2 School of Microelectronics, University of Chinese Academy of Sciences, Beijing 100049, China; 3 College of Life Sciences, Shanghai Normal University, Shanghai 200234, China; liweisimit@163.com

**Keywords:** piezoresistance, single-side micromachining, monolithic integration, tri-axis sensors, high-shock accelerometers

## Abstract

In this paper, a monolithic tri-axis piezoresistive high-shock accelerometer has been proposed that has been single-sided fabricated in a single (111)-silicon wafer. A single-cantilever structure and two dual-cantilever structures are designed and micromachined in one (111)-silicon chip to detect Z-axis and X-/Y-axis high-shock accelerations, respectively. Unlike the previous tri-axis sensors where the X-/Y-axis structure was different from the Z-axis one, the herein used similar cantilever sensing structures for tri-axis sensing facilitates design of uniform performance among the three elements for different sensing axes and simplifies micro-fabrication for the multi-axis sensing structure. Attributed to the tri-axis sensors formed by using the single-wafer single-sided fabrication process, the sensor is mechanically robust enough to endure the harsh high-*g* shocking environment and can be compatibly batch-fabricated in standard semiconductor foundries. After the single-sided process to form the sensor, the untouched chip backside facilitates simple and reliable die-bond packaging. The high-shock testing results of the fabricated sensor show linear sensing outputs along X-/Y-axis and Z-axis, with the sensitivities (under DC 5 V supply) as about 0.80–0.88 μV/g and 1.36 μV/g, respectively. Being advantageous in single-chip compact integration of the tri-axis accelerometers, the proposed monolithic tri-axis sensors are promising to be embedded into detection micro-systems for high-shock measurement applications.

## 1. Introduction

MEMS (microelectromechanical systems) inertial sensors can be widely used in the automobile industry and consumer electronics [1], among which high-shock sensors, i.e., high-*g* accelerometers with the measure range as tens of thousands of gravities, are essential to access and analyze structure destruction, rapture, and the collision process [2]. Cantilever-shaped piezoresistive high-g accelerometers were proposed for this kind of high-shock detection application [3]. 

Most of the previously reported cantilever high-shock sensing structures were fabricated with double-sided processes like the work in [4] where wet anisotropic etching from wafer backside creates difficulties in precise control of the deep etching depth. And the subsequent wafer-to-wafer bonding process introduces mismatching at the bonding interface, and thus, degrades the robustness of the sensor when it works in harsh high-shock detections. To solve the problem, a single-wafer single-side micromachined high-*g* sensor was proposed and developed for single-axis high-shock detection [5]. 

In many high-shock detection applications, however, tri-axis high-shock acceleration needs to be measured simultaneously. If three single-axis accelerometers are assembled together to take on the 3D high-shock detection task, the device will be quite large in volume, and any misalignment among the three axes will cause a decrease in the measurement accuracy. Apparently a monolithically integrated sensor has advantages in device miniaturization and precise axis alignment. However, the works on monolithic tri-axis high-g accelerometers have been seldom reported, with the accessed papers in [6,7,8]. The main difficulty of the monolithic tri-axis high-*g* accelerometers lies in the integration of the *Z*-axis sensing structure with the other two. In the works of [6] and [7], the fabricated structure of the out-of-plane Z-axis accelerometer was quite different from the other two in-plane sensing structures, where the X- and Y-axis sensing structures were in-plane deflective (or stretched) beams but the Z-axis accelerometer had to utilize a complicate twin-mass/tri-beam structure. The difference between the Z-axis structure and the X-/Y-axis one creates great difficulties in design of uniform performance between them. Similarly, the Z-axis accelerometer in [8] had to employ two seismic masses at the double sides of the X-/Y-axis structures. Not only the two masses occupied quite a large area of the chip, but also the two masses overhung out of the chip frame that really brought inconvenience to device packaging. Reference [8] did not provide testing results for the tri-axis sensor, and the device performance simulation results in [8] declared that the different Z-axis sensing structure from the other two caused considerable difference in sensing performance. Therefore, it is highly demanded to develop new micromachining techniques to form identical-shaped sensing structure, e.g., the same cantilever structures, for the monolithically integrated tri-axis sensor.

The research group of the authors developed a technique to fabricate in-plane deflective piezoresistive cantilevers only from the front-side of a single (111) silicon wafer for single-axis high-g accelerometers [5]. At that time, the vertically deflective Z-axis cantilever accelerometer could not be integrated with the in-plane cantilevers, and thus, monolithic tri-axis sensors could not be realized. In this study, tri-axis multi-cantilever high-shock accelerometers are eventually designed into monolithic integration and the fabrication is still performed simply from the single-side (front-side) of a single (111) wafer by developing a dual-step trench-etching based micromachining method. The fabricated tri-axis high-shock accelerometer chip is as small as 3.5 mm × 3.5 mm. High structure-design flexibility and high structure mechanical strength is ensured, attributed to the micromachining process. It is easy to attach the chip directly to most kinds of envelopes because the smooth backside is retained after the single-side process. Moreover, the single-sided MEMS processing method is compatible with standard IC-foundry that facilitates high-yield batch manufacturing. The design, fabrication, and testing of the single-chip integrated tri-axis sensor will be detailed in following sections.

## 2. Sensor Design

**Figure 1:**
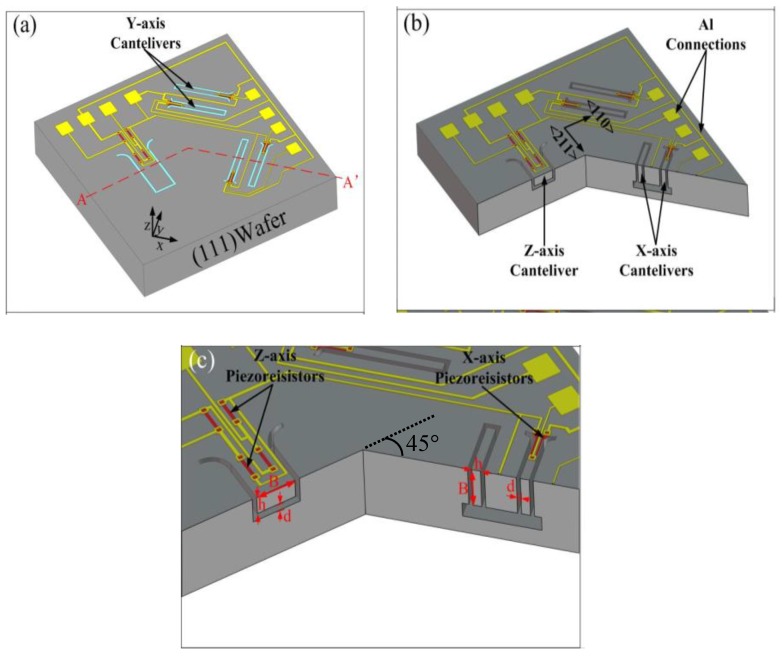
(**a**) 3D schematic of the tri-axis high-shock sensor chip. (**b**) Cross-sectional view of the chip cut along A-A’. (**c**) Magnified cross-sectional view of the Z-axis cantilever and X-axis cantilever.

Figure 1a shows the schematic view of the tri-axis sensor chip, whose size is 3.5 mm × 3.5 mm. The X-axis sensing structure is laid perpendicular to the Y-axis’s. The in-plane deflective sensing structure consists of two identical cantilevers which are put along opposite directions with each other. The Z-axis sensing structure is a vertically deflective cantilever, which has the same length as that of the X-/Y-axis ones. The longitudinal piezoresistive coefficients of P- type piezoresistor on (111) wafer are π_44_/2 and −π_44_/6 respectively and uniform along every orientations in the (111) plane [9], which facilitates flexible design and arrangement of the sensing piezoresistors. Thus the two cantilevers for the X-axis sensing unit are laid along the angle bisector of [110] and [211] orientations, and the other two cantilevers for Y-axis sensing (perpendicular to the X-axis ones) are along another angle bisector of [110] and [211]. The nest section will address that the anisotropic etching technique in (111) plane ensures that all the four cantilevers are formed exactly the same in structural dimensions. The Z-axis cantilever is arranged along [211] direction for the fastest structural formation by anisotropic etching release.

As is shown with the cutting line of A-A’ in Figure 1a, the cross section of the X-axis (the Y-axis is identical) and the Z-axis cantilevers are demonstrated in Figure 1b and the magnified view of Figure 1c. The denoted *B*, *h*, *l*, *d*, *l_p_*, *h*_0_ in Figure 1c represent the width, thickness, length, cantilever over-range protection gap, length of piezoresistor, and the distance from the piezoresistor neutral plane to the cantilever neutral plane, respectively. Theoretical analysis of mechanical bending of the cantilever under acceleration is performed to ensure robustness of the sensor, i.e., the maximum stress at the cantilever root is reliably lower than the rupture stress of single-crystalline silicon [10]. The sensitivity of the piezoresistive cantilever accelerometer with full Wheatstone bridge can be expressed as the following:

(1) $$S=\frac{\pi_{44}}{2}\times [\frac{2\rho gh_{0}}{h^{2}}(3l^{2}-3ll_{p}+l_{p}^{2})]\times V_{in}/g$$

The expression in the square bracket stands for the mean axial stress on each piezoresistor when 1 *g* acceleration perpendicular to the cantilever is applied. Herein *V_in_* is the input voltage supplied across the Wheatstone-bridge.

**Figure 2:**
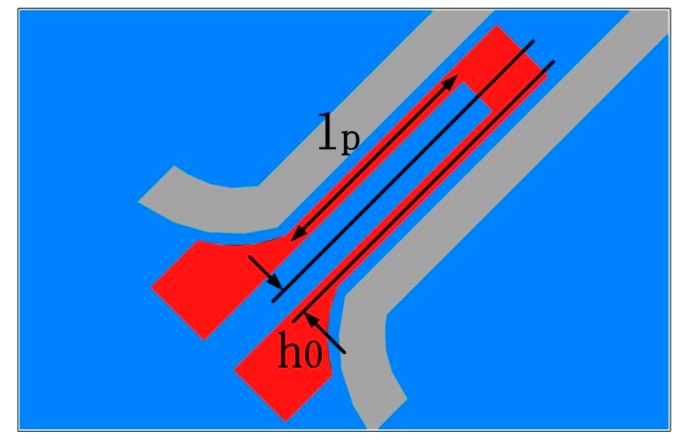
A piezoresistor located at the root of an X- or Y-axis cantilever. Herein *h*_0_ = 1/4*h* is designed.

The piezoresistors located on the Z-axis cantilever cannot be connected into full Wheatstone bridge, as transverse piezoresistive sensitivity in (111) plane and only longitudinal piezoresistive effect can contribute to the sensor. Thus, the two longitudinal piezoresistors are placed at the root of the Z-axis cantilever, where the two resistance values simultaneously increase or decrease along with out-of-plane deflection of the cantilever. Connected with another two fixed P-type resistors at the chip frame, a half sensitive Wheatstone bridge is configured. Two piezoresistors are located near the double side of each X-axis cantilever root, which are demonstrated in Figure 2. One resistance will increase and the other will decrease when the X-axis cantilever laterally deflects under in-plane high-shock acceleration.

In the Z-axis accelerometer with half Wheatstone bridge, *h*_0_ = *h*/2 is designed; however, in the X-/Y-axis accelerometer with full Wheatstone bridge, *h*_0_ = *h*/4 is designed. Therefore, the sensitivity expression for each of the three axes accelerometers can be rewritten as the following:

(2) $$S=\frac{1}{2}\times \frac{\pi_{44}}{2}\times [\frac{\rho g}{h}(3l^{2}-3ll_{p}+l_{p}^{2})]\times V_{in}/g$$

If the cantilever dimension values of *h, l, l_p_* are the same for each axis, the sensitivities for every axis will be identical. The resonant frequency of the cantilever can be expressed as the following:

(3) $$f=\frac{1.019}{2\pi }\times \frac{h}{l^{2}}\times \sqrt{\frac{E}{\rho }}$$

The damping coefficients for Z and X-/Y-axis cantilevers can be calculated by the following:

(4) $$\zeta_{z}=\beta \frac{\mu B^{2}L^{2}}{d^{3}h^{2}}\sqrt{\frac{1}{\rho E}}$$

(5) $$\zeta_{x/y}=2\beta \frac{\mu B^{2}L^{2}}{d^{3}h^{2}}\sqrt{\frac{1}{\rho E}}$$

where *β* is the correction factor, which is a function of *B/l*, and can be read in the data from [10]. In order to achieve adequate damping coefficient, *d* should be small enough, and the width of the cantilevers *B* should be much larger than the thickness *h*. For balancing the air squeeze film damping effect among different cantilevers, the relevant cantilever dimensions for individual cantilevers are optimally designed, as are listed in Table 1. The designed sensitivity and resonant frequency for all the three axes are 0.131 μV/V/g (i.e., 0.66 μV/g under 5V supply on the Wheatstone bridge) and 134.7 kHz.

The high-shock acceleration measure range *a_r_* is limited by *d,* which is the maximum possible deflection of the cantilever, and thus expressed with the following:

(6) $$a_{r}=\frac{2dEh^{2}}{3\rho L^{4}}$$

From another view point, the maximum deflection induced maximum stress at the cantilever root is equal to 3*ρal^2^/h*, which should not be larger than the rupture stress of a single-crystalline-silicon micromechanical structure of about 300 MPa [10]. Therefore the acceleration measure range for each of the three cantilever sensor has the potential to be as high as 390,000 *g*. Of course, the device packaging should be strong enough to meet the reliability requirement of high-g measurement.

Cross-axis interference is an important concern of the tri-axis accelerometer and the cross-axis response to the acceleration along the cantilever-width direction (but perpendicular to the main sensing direction) is the key consideration. When Z-axis acceleration is exerted on the X- or Y-axis sensor, the response sensitivity can be well suppressed, since identical bending stress is exerted on the two sensitive piezoresistors in each cantilever. The common sensing signal can be cancelled out by the balance effect of the Wheatstone bridge. Moreover, the large cantilever width/thickness ratio of 50 μm/18 μm further decreases the generated cross-axis stress signal. For the Z-axis cantilever under in-plane transverse cross-axis acceleration, however, the two piezoresistors do generate differential piezoresistive signals. Fortunately, the much larger width/thickness ratio of 180 μm/18 μm secures very low cross-axis interference. 

## 3. Fabrication

**Figure 3:**
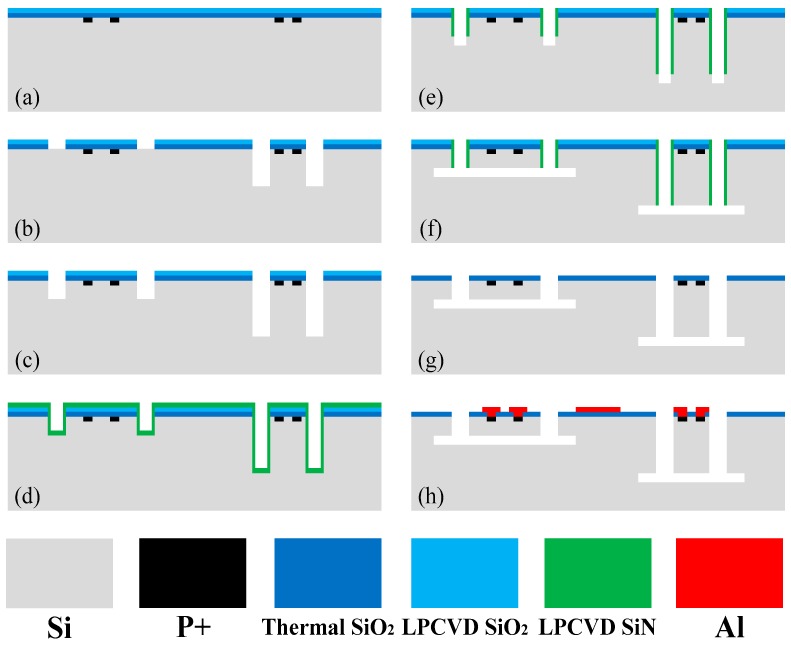
Single-side process steps for the tri-axis shigh-shock accelerometer.

The single-side fabrication process-flow is showed in Figure 3 and step-by-step described as follows: The cross-section views are cut along the line of A-A’ in Figure 1a to show the Z-axis cantilever (at left) and the X-axis cantilever (at right) together under every micromachining cantilever-formation steps. The fabrication for the piezoresistors is combined together for showing more process details, though the piezoresistors are not located at the cutting line of A-A’.

(a) N-type (111) 4-inch silicon wafers, with resistivity as 3∼10 Ω·cm and thickness as 450 μm, is thermally oxidized. Then the oxide layer is patterned with photolithography and etched by buffered HF to expose the piezoresistor areas. After boron ion implantation and diffusion, the target impurity concentration is about 1 × 10^19^ atoms/cm^3^ to satisfy both piezoresistive sensitivity and Ohmic contact with following metal interconnection lines. The target square resistance is about 100 Ω. Then a LPCVD (low-pressure chemical vapor deposition) oxide layer is deposited.
(b) The relatively shallower trench etching window for the Z-axis cantilever is opened with photolithography and reactive ion etch (RIE). Then, the relatively deeper etch windows for the X-/Y-cantilevers are opened by photolithography and RIE, during which the photoresist covering the Z-cantilever is retained for protection from etched. Then silicon deep DRIE is conducted only at the windows for X-/Y-cantilevers to the depth of *B*_x_*-h*_z_, which equals to the difference of depth between the width of the lateral deflective X-/Y-cantilevers and the thickness of the vertical deflective Z-cantilever.
(c) After the photoresist is removed, the second-step deep RIE is processed to reach the depth of *h*_z_ through both the window of Z-cantilever and those for the X-/Y-cantilevers. In this way the width of the X-/Y-cantilevers and the thickness of the Z-cantilever can be simultaneously obtained as *B*_x_ and *h*_z_, respectively
(d) LPCVD low-stress silicon nitride is deposited to cover all the trench bottoms and the side-walls.
(e) The silicon nitride at the bottom of the trenches is removed by RIE, while the silicon nitride at the sidewalls is retained. Then, deep RIE is conducted to form a further depth from the exposed bottom silicon. The depth is equal to the designed squeeze-film air damping gap of the Z-cantilever.
(f) All the cantilevers are released by anisotropic wet etching with 20% aqueous TMAH (tetramethylammonium hydroxide) at 90 °C. The etching starts from the silicon-exposed bottom segment of the trench. The etching is processed laterally, with the (111) ceiling and floor almost untouched. The (111) etching properties can be found in [11].
(g) The remained siliconnitride and silicon oxide are removed by 85% H_3_PO_4_ at 165 °C and HF, respectively.
(h) Contact holes are opened and Al is sputtered and patterned by ion-beam etch to form interconnection lines.

**Figure 4:**
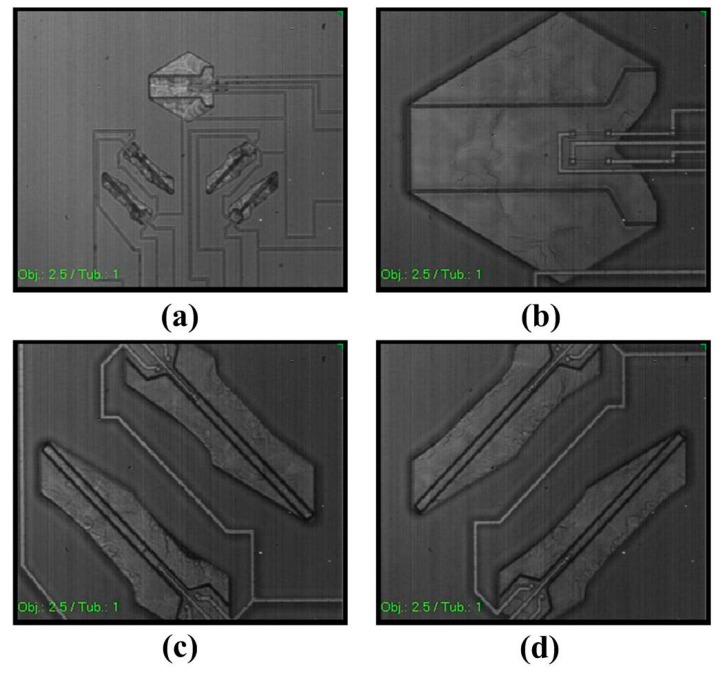
(**a**) infrared microscopic image showing the laterally etched cavities under every cantilever. (**b–d**) Cavities under the Z-cantilever, X-axis cantilevers, and Y-axis cantilevers, respectively.

The lateral anisotropic-etch formed cavities under the five cantilevers are showed in the infrared microscopic images of Figure 4a, where the Z-cantilever cavity is very clear but the X-/Y-cantilever cavities are a little obscure, due to the cavities not being at the same depth for the infrared-light focusing, and the infrared light passes through relatively thicker layers of silicon for the X-/Y-cantilever cavities. As is showed in Figure 4b, the cavity under the Z-axis cantilever is as large as about 500 μm × 500 μm, which needs to be released by a somewhat longer time of anisotropic etch. The cavities under the X-axis cantilevers (see Figure 4c) are exactly the same as those under the Y-axis cantilevers (see Figure 4d), except that the direction is rotated by 90°. The X-/Y-cantilevers are with the longitudinal direction along the angular bisector of 〈110〉 and 〈211〉, i.e., $\langle -2,1-\sqrt{3},1+\sqrt{3}\rangle$ or $\langle -2,1-\sqrt{3},1+\sqrt{3}\rangle$. Thanks to the etching atop properties in the (111) wafer, the etching time tolerance is large enough to complete the structure release of the tri-axis cantilevers with satisfactory fabrication yield.

**Figure 5:**
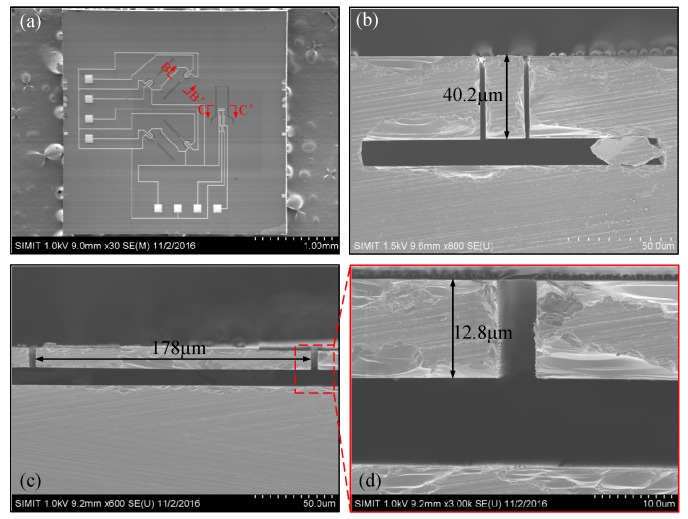
(**a**) Top-view SEM image of the whole tri-axis sensor chip. (**b**) Cross-sectional view of the X-/Y-axis cantilever cut along along B-B’ in (**a**). (**c**) Cross-sectional view of the Z-axis cantilever cut along C-C’ in (**a**). (**d**) Magnified cross-sectional locaiton of the Z-axis cantilever.

The fabricated tri-axis high-shock accelerometer chip (3.5 mm × 3.5 mm) is showed in the top-view SEM image of Figure 5a. Four narrow cantilevers for X-/Y-axis sensing and one wide cantilever for vertical Z-axis sensing are formed in one chip. There are two aluminum pads used for input voltage and six pads for output voltage of the three axes. The cross section of the fabricated X-/Y-cantilevers cut along B-B’ is showed in Figure 5b. The cross section of the fabricated Z-cantilever cut along C-C’ is showed in Figure 5c, with a part further magnified in Figure 5d. It is worth pointing out that the etching time is too long (for avoiding incomplete release of the Z-cantilever) and, in this case, the very slow etch to the (111) plane cannot be thoroughly neglected. About 5 μm thickness of the finally formed Z-cantilever is over etched off. The difference of the fabricated device dimensions from the design would cause deviation in sensitivity to shocking acceleration.

## 4. Testing Results

**Figure 6:**
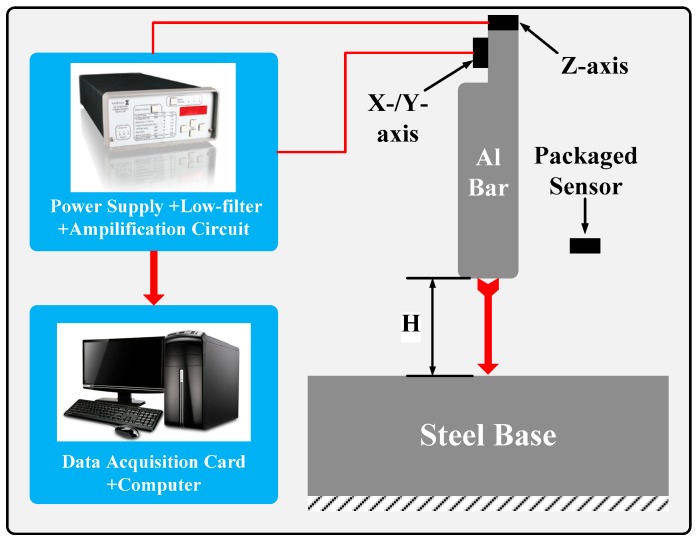
Schematic of the dropping hammer testing system.

The fabricated tri-axial dual-cantilever high-shock accelerometer is packaged in a ceramic envelop and tested with a dropping hammer system [12], where an Endevco-136 instrument is used to provide a DC 5 V power supplier, a switchable low-pass filter (with the bandwidth as 10 kHz) and a variable gain amplifier. The packaged sensor is fixed on one end of a stainless steel bar, which is dropped freely from a desired height to collide with a fixed steal base on the ground, as is schematically shown in Figure 6. A high-shock signal is generated by the approximately elastic collision between the free dropping bar and the fixed block, and the dynamic response voltage of the accelerometer is picked up by a data acquisition interface and then analyzed by data processing software in a personal computer.

**Figure 7:**
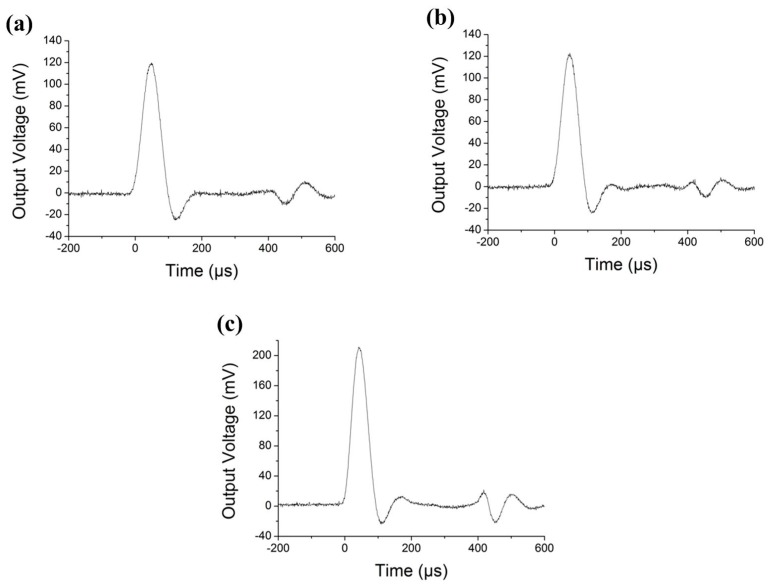
High-shock testing results by dropping the steel-bars from a height of 25 cm. (**a–c**) show the outputs of the X-, Y- and Z-axis sensing units, which are measured for calculating the sensitivities.

With the low-pass filter switched on, Figure 7a–c show the transient high-shock responses of the X-, Y- and Z-axis accelerometer units to the collision by dropping the bar from a height of 25 cm that generates under 8000 *g* acceleration (calibrated by a commercial PCB-301A12 standard accelerometer, Tektronix, Beaverton, OR, USA). The downwards pulse just after the main collision pulse is caused by the rebound up process of the bar after collision. The X-/Y-axis accelerometers exhibit almost the same sensitivity of 0.80–0.85 μV/g, and the Z-axis accelerometer shows 1.36 μV/g sensitivity under the 8000 *g* shock. Then the low-pass filter is switched off and the response signals are processed by Fourier transformation to obtain the power spectrum density within the bandwidth. The resonant frequencies of the X- Y- and Z-axis sensors are measured as 112 kHz, 117 kHz and 76 kHz, respectively. Though the relationship between dropping height and the generated high-shock acceleration of the dropping bar testing system is calibrated by using the commercial standard accelerometer, the measurement error has been estimated at about 5–7%.

The measured sensitivities and resonant-frequencies of the X- and Y-axis units generally agree with design. The slightly larger measured sensitivity than design mainly comes from the underestimation to the designed piezoresistive coefficient of π_44_, which depends on the doped boron concentration of the piezoresistors. However the Z-axis sensitivity is much higher than design and its resonant frequency is much lower. The reason lies in the over etched Z-axis cantilever. Being obtained by measuring the process dummy wafer under SEM, the Z-axis cantilever thickness is about 5 μm smaller than the 18 μm of the X- or Y- axis cantilever. Equation (2) shows that the sensitivity is inversely proportional to the cantilever thickness. Therefore the Z-axis sensitivity should be about 18/13 times of the X-/Y-axis sensitivity, i.e. about 1.11–1.22 μV/g that is close to the measured 1.36 μV/g. Exhibiting a higher sensitivity than design, the much thinner Z-axis cantilever will do feature a lower resonant frequency than design (see Equation (3)).

With the designed cantilever structures for sensing along the three axes, the sensitivities and resonant frequencies for the three-axis sensing units could be formed very close with each other, as long as the fabricated Z-axis cantilever thickness is almost the same as that of the X-/Y-axis cantilevers. In contrast, for the previous works in [6,7,8] where the Z-axis sensing structure is quite differently configured and designed from that of the X-/Y-axis ones, uniform tri-axis sensitivity and resonant frequency cannot be simultaneously obtained.

**Figure 8:**
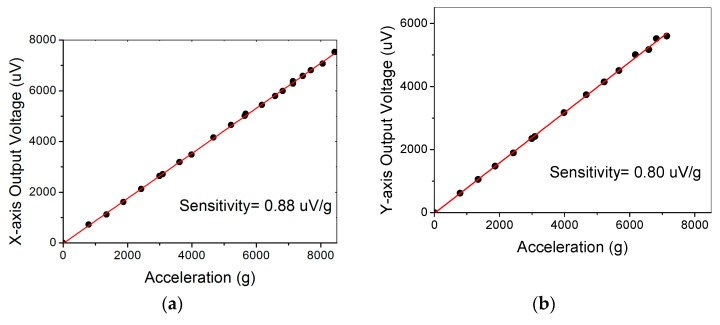

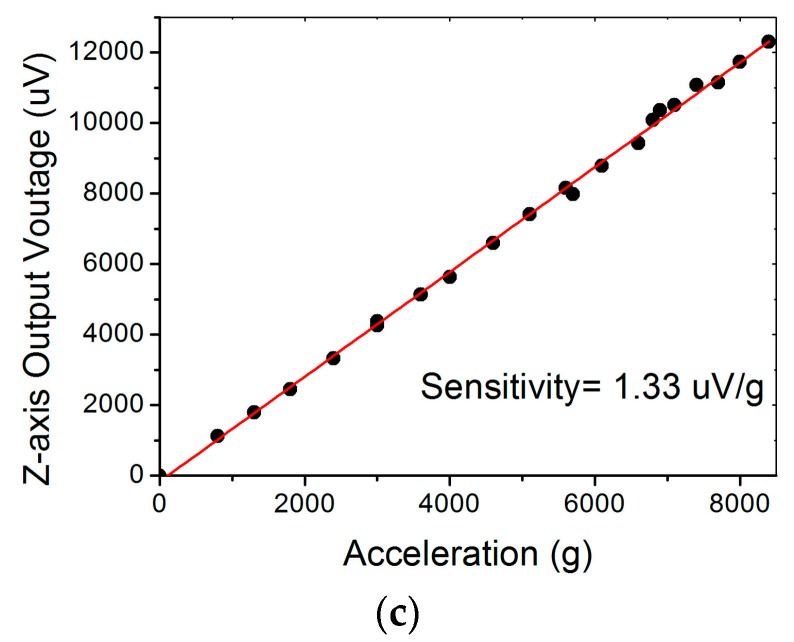
Testing results of the X-axis sensor, Y-axis sensor, and the Z-axis sensor, respectively in (**a–c**), by using a B&K 9525C compressing-air shock generating system.

The packaged tri-axis sensor is then tested by using a commercial B&K 9525C compressing-air shock generating system, where the high-g acceleration is generated by compressing the N_2_ gas and then suddenly releasing the high-pressure chamber to accelerate a hammer to collide with a base. In this way the shock processing period of time is a bit longer than that from the dropping bar system. The measurement error of the system is given in the handbook as smaller than 5%. All the three-axis sensing unites are with the testing results showed in Figure 8. The measured sensitivities are very close to the results obtained from the dropping hammer system.

## 5. Conclusions

A monolithic tri-axis micro-cantilever high-shock accelerometer is proposed and developed. The micromachining fabrication is implemented in a single (111) silicon wafer and the process is only from the front-side of the wafer, which is compatible with the IC-foundry batch-fabrication scheme. The multi-axis sensor consists of two couples of in-plane deflective piezoresistive cantilevers for X-axis and Y-axis sensing and an out-of-plane deflective cantilever for Z-axis sensing. This configuration facilitates designing uniform sensitivity and resonant frequency for the three sensing elements. A dynamic shock test of the sensor is performed by using a dropping bar system, with the results verifying that this tri-axis accelerometer can be used to measure high-g shock in the X-, Y-, and Z-axis. The sensitivity testing results are also confirmed by using a compressing-air hammer shock generating system. The sensitivity of the Z-axis sensing unit is a bit larger than that of the X-/Y-axis ones due to the fabricated Z-axis single-cantilever being 5 μm thinner than design, and can be rectified by modifying the depths of the two-step deep RIE trench. The results show that the developed monolithic tri-axis sensors are expected to be applied in high-shock detection systems.

## Figures and Tables

**Table 1:** Designed dimensions of the cantilevers.

Symbol	Quantity	Value
*l*	length of cantilevers	445 μm
*h*	thickness of cantilever	18 μm
*l_p_*	length of piezoresistors	60 μm
*B*_Z_	width of Z-axis cantilever	180 μm
*d*_Z_	gap of Z-axis cantilever	5 μm
*B*_XY_	width of X/Y-axis cantilevers	50 μm
*d*_XY_	gap of X/Y-axis cantilevers	2.5 μm
